# Multi-contrast brain magnetic resonance image super-resolution using the local weight similarity

**DOI:** 10.1186/s12880-016-0176-2

**Published:** 2017-01-17

**Authors:** Hong Zheng, Xiaobo Qu, Zhengjian Bai, Yunsong Liu, Di Guo, Jiyang Dong, Xi Peng, Zhong Chen

**Affiliations:** 1 Department of Electronic Science, Fujian Provincial Key Laboratory of Plasma and Magnetic Resonance, Xiamen University, Xiamen, 361005 China; 2 School of Computer Science and Engineering, Key Laboratory of Intelligent Processing of Image and Graphics, Guilin University of Electronic Technology, Guilin, 541004 China; 3 School of Mathematical Sciences, Xiamen University, Xiamen, 361005 China; 4 School of Computer and Information Engineering, Fujian Provincial University Key Laboratory of Internet of Things Application Technology, Xiamen University of Technology, Xiamen, 361024 China; 5 Paul C. Lauterbur Research Centre for Biomedical Imaging, Shenzhen Institutes of Advanced Technology, Chinese Academy of Sciences, Shenzhen, Guangdong 518055 China

**Keywords:** Super-resolution, Multi-contrast, Statistical information, Weight, Non-iterative process

## Abstract

**Background:**

Low-resolution images may be acquired in magnetic resonance imaging (MRI) due to limited data acquisition time or other physical constraints, and their resolutions can be improved with super-resolution methods. Since MRI can offer images of an object with different contrasts, e.g., T1-weighted or T2-weighted, the shared information between inter-contrast images can be used to benefit super-resolution.

**Methods:**

In this study, an MRI image super-resolution approach to enhance in-plane resolution is proposed by exploring the statistical information estimated from another contrast MRI image that shares similar anatomical structures. We assume some edge structures are shown both in T1-weighted and T2-weighted MRI brain images acquired of the same subject, and the proposed approach aims to recover such kind of structures to generate a high-resolution image from its low-resolution counterpart.

**Results:**

The statistical information produces a local weight of image that are found to be nearly invariant to the image contrast and thus this weight can be used to transfer the shared information from one contrast to another. We analyze this property with comprehensive mathematics as well as numerical experiments.

**Conclusion:**

Experimental results demonstrate that the image quality of low-resolution images can be remarkably improved with the proposed method if this weight is borrowed from a high resolution image with another contrast.

**Graphical Abstract:**

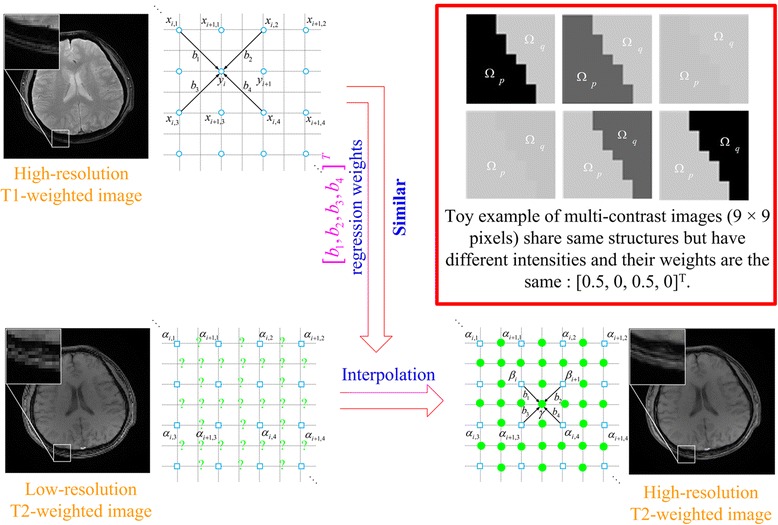

Multi-contrast MRI Image Super-resolution with Contrast-invariant Regression Weights

**Electronic supplementary material:**

The online version of this article (doi:10.1186/s12880-016-0176-2) contains supplementary material, which is available to authorized users.

## Background

In MRI, low-resolution (LR) images may be acquired in applications, e.g., functional MRI [1, 2] and diffusion tensor imaging [3, 4], due to limited data acquisition time or other physical constraints. High-resolution (HR) images appear favorable to perform subsequent posterior image processing and visualization [5]. Super-resolution methods are widely utilized to improve image resolution [6–10]. Typical methods include sparse representations [6–8], projection onto convex sets (POCS) [9], tensor frames [10], etc. However, these methods need numerous iterations to accomplish super-resolution, thus they inevitably lead to high computational costs. For MRI, since a great number of images have to be processed, fast and stable methods are desired. Recently, the prior information of MRI has been explored in super-resolution. For example, (a) redundant information produced by sub-pixel spatial shifts between multiple images [3], (b) space homogeneity constraint from orthogonal anisotropic acquisitions [2], and (c) the learned dictionary with a nature of the orthogonality [11] have been employed to refine structural details and edges. Besides, image contrast can also be utilized to produce sharper images [12]. However, these methods may not lead to faithful super-resolution results when multiple-shifted images are inapplicable or the information is very limited within a single image. Thus, one may expect other prior information beyond a single image.

Multi-contrast images are frequently acquired in MRI experiments [13]. For example, plentiful edge structures are visible both in T1-weighted and T2-weighted brain images of the same subject. According to the principles of MRI [14], we pick up T1 or T2 weighted signal denoted by *SI* and take the form

1 $$ SI\propto \kern0.7em \rho (H)\left(1-{e}^{-\mathrm{T}\mathrm{R}/\mathrm{T}1}\right)\left({e}^{-\mathrm{T}\mathrm{E}/\mathrm{T}2}\right) $$

where *ρ*(*H*) refers to the proton density, TR is the repetition time and TE is the echo time. There are different TR value and TE value within a section of medical tissue that would result in multiple contrast images. Yet, these images share the proton density of the subject so that they largely share similar anatomical structures but with different contrasts in regions. The shared information between inter-contrast images can be considered to benefit super-resolution. Therefore, it is possible to improve the LR image resolution by incorporating prior information from the different contrast image in HR. Rousseau proposed a patch-based iterative framework combining with non-local similarity to share information among multiple contrast images in [15], and later many more detailed analysis was studied in [16]. A constraint that the downsampled version of the reconstructed LR data must be equal to the original LR data is imposed in the iterative framework [5]. The non-local similarity is also measured with both voxel intensity and gradient intensity in super-resolution [17]. However, these methods require training sets or time-consuming iteration processing.

New edge-directed interpolation (NEDI) [18] is a fast and statistical super-resolution method for a single image. It estimates local covariance coefficients from a LR image and assumes that this statistical information is also valid for the corresponding HR image. A pixel of the HR image is interpolated by performing the linear regression of neighboring pixels, which originate from the LR image. This regression process is based on non-iterative operations, thus the super-resolution can be performed fast. The NEDI provides a nice way of analyzing statistical information in the image super-resolution. Some recent methods [19–21] also use regressions to improve the image resolution and achieve remarkable performances. However, these methods train hundreds of external images prior to recovering structural details, and require plenty of computations. Due to the nice statistical property and low computation time of NEDI, in this work, we extend it into the multi-contrast image super-resolution and demonstrate its superior performance on MRI images.

We will explore how to incorporate the statistics from one image into another contrast image. Regression weights, estimated from a HR image in one contrast, and neighboring pixels around the interpolated location in the LR image of another contrast work together to generate a new pixel value. The fact that neighbors are provided by the LR image itself can offer a guarantee and support for the consistent contrast between the LR one and the interpolated result. Mathematical analysis and experimental evidence will be presented to address a fundamental question of why these weights between two contrast images constitute faithful criteria. Then, the proposed approach probes the information both from a LR image and its corresponding HR image in another contrast. Our method will be compared with the classic bicubic method, NEDI method [18], and the state-of-the-art contrast-guided interpolation (CGI) method [12] in terms of objective-evaluation criteria and visual perceptions.

The remainder of this article is organized as follows: In section II, we briefly review basic concepts of NEDI. In section III, we derive conditions that must be satisfied in our method. Experimental results and discussions will be presented in sections IV. Finally, concluding remarks are made in section V.

## Method

### Brief review of NEDI

In NEDI, regression weights are estimated in a local region then target pixels are calculated as a linear regression of neighbors [18]. Thus, it is crucial to determine the regression weights in the interpolation. Within a neighborhood, four neighbors are commonly used in NEDI, and consequently there are four regression weights for one pixel interpolation.

**Fig. 1:**
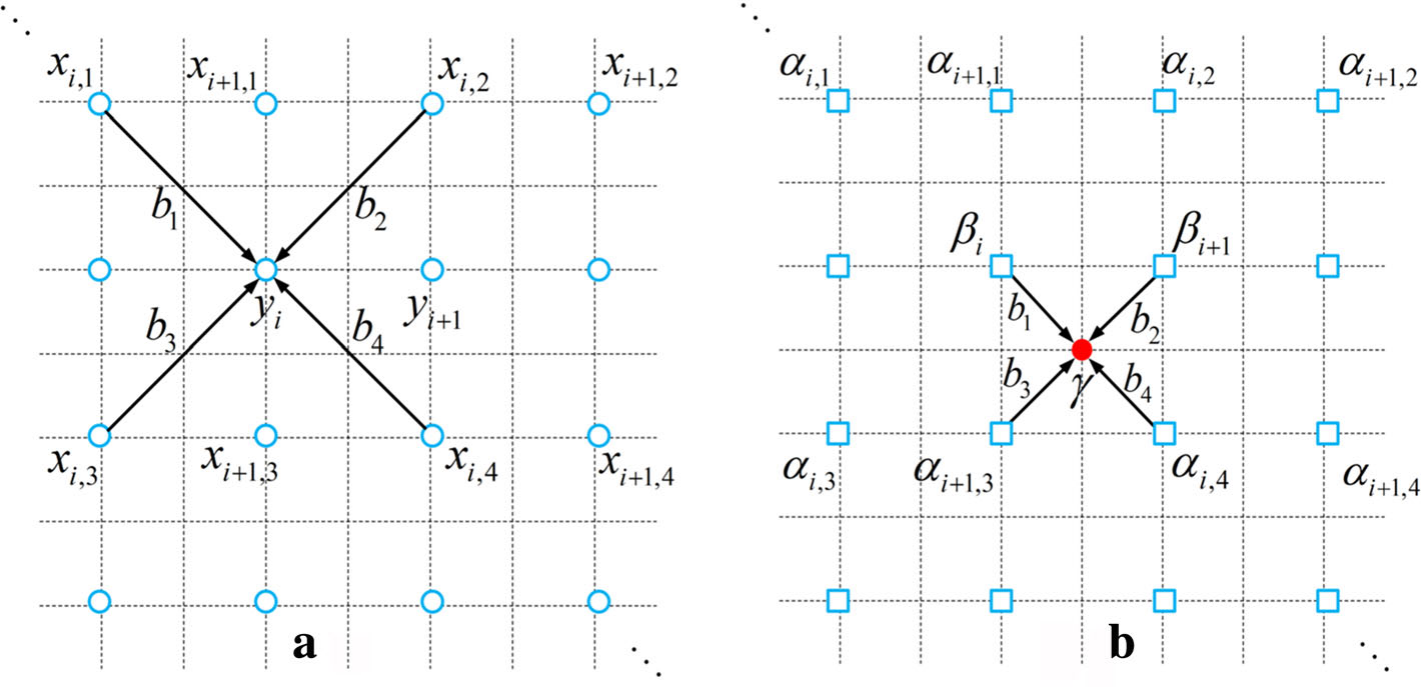
Interpolation process in NEDI. **a** Generating regression weights by 1-pixel-width overlap patches (with moving from left to right and from top to bottom) inside a local region; **b** Interpolating a new pixel *γ* by multiplying neighbors and 4 regression weights estimated from (**a**)

The interpolation process is shown in Fig. 1. The NEDI uses patches in the local region to estimate regression weights *b*_*j*_(*j* = 1, 2, 3, 4) (Fig. 1a). The variable *n* (*i* = 1, ⋅ ⋅ ⋅, *n*) denotes the number of patches and each patch is composed of one pixel *y*_*i*_ and its four neighbors *x*_*i*,*j*_ along diagonal directions. Then, the target pixel *γ* is obtained by multiplying neighbors and their weights (Fig. 1b).

The basic regression model (Fig. 1a) applied in our work is

2 $$ {y}_i={b}_1{x}_{i,1}+{b}_2{x}_{i,2}+{b}_3{x}_{i,3}+{b}_4{x}_{i,4}+\kern0.3em {\varepsilon}_{i\kern0.1em }\kern0.1em , $$

where *ε*_*i*_ is the residual error. By continually sampling in a 9 × 9 region, a vector **y** = [*y*_1_, ⋯, *y*_49_]^*T*^ ∈ *ℝ* ^49^ is formed to represent pixels in this region and meanwhile a matrix **X** = [**x**_1_, ⋯, **x**_49_] ∈ *ℝ* ^49 × 4^, whose column **x**_*i*_ contains four neighbors of *y*_*i*_, is formed to represent all neighboring pixels around those pixels of **y**.

Assuming the image pixel values in a local region satisfy a locally stationary Gaussian process [18], the regression weight $$ \mathbf{b}={\left[\begin{array}{cccc}\hfill {b}_1\hfill & \hfill {b}_2\hfill & \hfill {b}_3\hfill & \hfill {b}_4\hfill \end{array}\right]}^T $$ is estimated according to

3 $$ \underset{\mathbf{b}}{ \min }{\left\Vert \mathbf{y}-\mathbf{X}\mathbf{b}\right\Vert}_2, $$

and its solution is

4 $$ \mathbf{b}={\left({\mathbf{X}}^T\mathbf{X}\right)}^{-1}\left({\mathbf{X}}^T\mathbf{y}\right). $$

The above analysis can be also interpreted from the classical Wiener filtering theory. Let **R** = (**X** ^*T*^ **X**)^− 1^ ∈ *ℝ* ^4 × 4^ represents a covariance between two arbitrary members of the four nearest neighbors, **r** = **X** ^*T*^ **y** ∈ *ℝ* ^4^ represents a covariance between the center-pixel and the one of the four nearest neighbors around it, the optimal coefficients can be found by

5 $$ \mathbf{b}={\mathbf{R}}^{-1}\mathbf{r}. $$

### Multi-contrast image super-resolution

In the proposed method, a HR image of one contrast is assumed to be available for interpolating a LR image of another contrast. This assumption is reasonable since multi-contrast images are always available in MRI experiments [5, 7, 13].

The regression weights **b**_*i*_ for the *i* ^th^ pixel, borrowed from one contrast HR image according to Eq. (4), is incorporated into the interpolation of the LR image in another contrast. Interpolated pixels ỹ_*i*_ of an expected HR image are given by

6 $$ {\tilde{y}}_i={{\mathbf{b}}_i}^T{\mathbf{s}}_i $$

where the vector **s**_*i*_ includes four pixels of the LR image that are the nearest neighbors along diagonal directions of the *i* ^th^ pixel in the center. This means we assume that the HR image in Fig. 1a is in one contrast and the LR image in Fig. 1b is in another contrast. Then **b**_*i*_ is estimated from Fig. 1a and **s**_*i*_ comes from Fig. 1b. Therefore, this new approach absorbs prior information from the HR image in one contrast and maintains the data consistency of LR image in another contrast.

To facilitate following discussion, intensities of images are all normalized between 0 and 1. Furthermore, we assume that multi-contrast images are well registered before super-resolution.

#### Weights in multi-contrast images

**Fig. 2:**
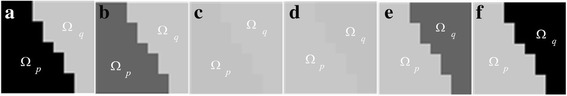
A toy example of multi-contrast images of size 9×9. **a-f** share the same structure but have different intensities

For example, multi-contrast images (Fig. 2) share similar anatomical structures but are with different intensities in sub-regions.

**Fig. 3:**
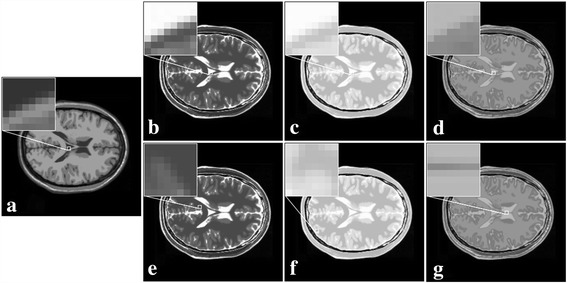
Sub-regions with same or different anatomical structures in synthetic MRI images in (**a**-**g**)

An interesting phenomenon is that, regression weights for different contrast images in Fig. 2 are nearly the same (Table 1). The same observation is also found (Table 2) for MRI images generated from the BrainWeb [22] that embody more complex structures (Figs. 3a–d). However, regression weights (Table 3) will be totally different if images do not share the similar anatomical structures (Figs. 3a, e–g). These observations convey important information: The regression weights obtained using the least square estimation is nearly invariant to image contrasts. If this is possible, one may easily employ the information from another contrast image by making use of these weights.

**Table 1:** Regression weights for synthetic images shown in Fig. 2

	Fig. 2a	Fig. 2b	Fig. 2c	Fig. 2d	Fig. 2e	Fig. 2f
( Ω_*p*_, Ω_*q*_)	(0, 0.78)	(0.39, 0.78)	(0.76, 0.78)	(0.78, 0.76)	(0.78, 0.39)	(0.78, 0)
**b**	[0.50;0.00; 0.00; 0.50]	[0.50;0.00; 0.00; 0.50]	[0.50;0.00; 0.00; 0.50]	[0.50;0.00; 0.00; 0.50]	[0.50;0.00; 0.00; 0.50]	[0.50;0.00; 0.00; 0.50]
∑_*j* = 1_^4^ *b*_*j*_	1.00	1.00	1.00	1.00	1.00	1.00

**Table 2:** Regression weights in regions of zoom for same anatomical structures shown in Fig. 3

	Fig. 3a	Fig. 3b	Fig. 3c	Fig. 3d
**b**	[−0.19;0.70;0.55;−0.07]	[−0.15;0.68;0.53;−0.06]	[−0.18;0.68;0.59;−0.09]	[−0.05;0.56; 0.53;−0.04]
∑_*j* = 1_^4^ *b*_*j*_	0.99	1.00	1.00	1.00

**Table 3:** Regression weights in regions of zoom for different anatomical structures shown in Fig. 3

Images	Fig. 3a	Fig. 3e	Fig. 3f	Fig. 3g
**b**	[−0.19;0.70; 0.55;−0.07]	[0.44;0.04; 0.03;0.49]	[0.22;0.23; 0.33;0.21]	[−0.95;1.45; −0.72;1.21]
∑_*j* = 1_^4^ *b*_*j*_	0.99	1.00	0.99	0.99

Besides, one may find that the sum of weights in each vector is approximately 1 (Tables 1, 2 and 3). We will analyze this property with comprehensive mathematics and empirical tests on MRI images. This property will be an important foundation to derive similar regression weights for multi-contrast images.

#### Sum of weights is approximately equal to 1

Suppose there are *n* central pixels, by adding *n* operations in a local region, Eq. (2) is written as

7 $$ {\displaystyle {\sum}_{i=1}^n{y}_i}={b}_1{\displaystyle {\sum}_{i=1}^n{x}_{i,1}}+{b}_2{\displaystyle {\sum}_{i=1}^n{x}_{i,2}}+{b}_3{\displaystyle {\sum}_{i=1}^n{x}_{i,3}}+{b}_4{\displaystyle {\sum}_{i=1}^n{x}_{i,4}}+{\displaystyle {\sum}_{i=1}^n{\varepsilon}_{i\kern0.1em }}\kern0.2em . $$

Here, *ε*_*i*_ is assumed to satisfy the normal distribution, i.e., *ε*_*i*_ ~ *N*(*μ*_*i*_, *σ* ^2^). The variable *μ*_*i*_ is the mean and *σ* ^2^ is the variance associated with *ε*_*i*_. Then we can easily have ∑_*i* = 1_^*n*^ *ε*_*i*_ = ∑_*i* = 1_^*n*^ *μ*_*i*_ + ∑_*i* = 1_^*n*^ *ε*′, where there exists *ε*_*i*_^′^ ∼ *N*(0, *σ* ^2^). Next, according to the principle of the law of large number, meaning that sufficient central pixels are sampled, one has

8 $$ {\displaystyle {\sum}_{i=1}^n{y}_i}={b}_1{\displaystyle {\sum}_{i=1}^n{x}_{i,1}}+{b}_2{\displaystyle {\sum}_{i=1}^n{x}_{i,2}}+{b}_3{\displaystyle {\sum}_{i=1}^n{x}_{i,3}}+{b}_4{\displaystyle {\sum}_{i=1}^n{x}_{i,4}}+{\displaystyle {\sum}_{i=1}^n{\mu}_i}\kern0.1em , $$

where ∑_*i* = 1_^*n*^ *μ*_*i*_ is a fairly small constant. Then, given that ∑_*i* = 1_^*n*^ *x*_*i*,1_, ∑_*i* = 1_^*n*^ *x*_*i*,2_ , ∑_*i* = 1_^*n*^ *x*_*i*,3_ , ∑_*i* = 1_^*n*^ *x*_*i*,4_ and ∑_*i* = 1_^*n*^ *y*_*i*_ are equal to one another, one can obtain that the sum of weights follows

9 $$ {\displaystyle {\sum}_{j=1}^4{b}_j}\approx 1. $$

**Fig. 4:**
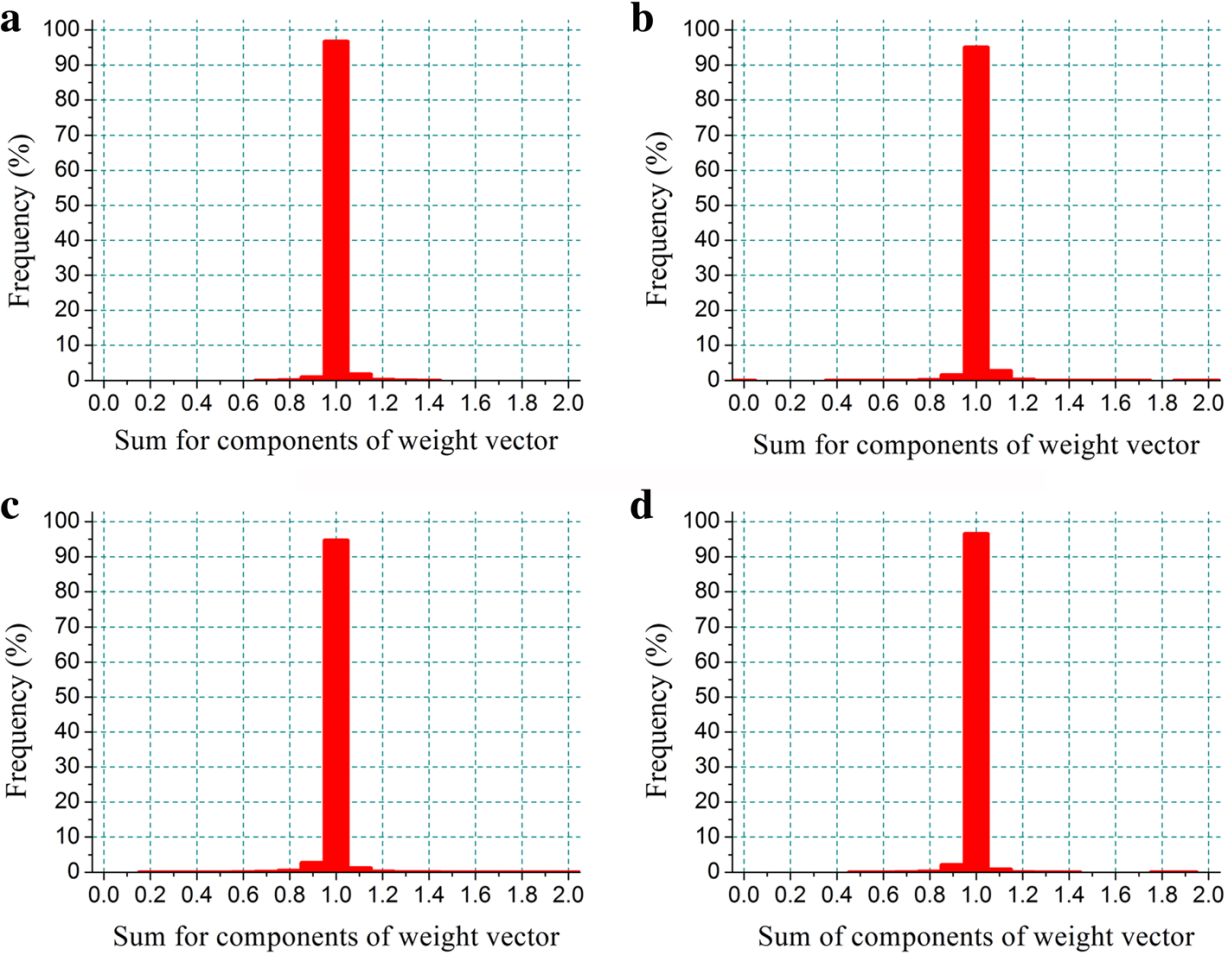
Sum of weights (i.e., ∑_*j* = 1_^4^ *b*_*j*_) on MRI data. The vertical axis represents the percentage that the estimation values of ∑_*j* = 1_^4^ *b*_*j*_ lies in the corresponding values in the horizontal axis. **a**-**b** list the frequency of ∑_*j* = 1_^4^ *b*_*j*_ for simulated images (i.e., Fig. 3a and b). **c**-**d** list the frequency of ∑_*j* = 1_^4^ *b*_*j*_ for real images (i.e., Fig. 9a and b)

We verify this property that sum of weights is approximately equal to 1 on MRI images. Statistical analysis in Fig. 4 show that most of ∑_*j* = 1_^4^ *b*_*j*_ are very close to 1 for tested images. In each image, the range of ∑_*j* = 1_^4^ *b*_*j*_ lies between 0.95 and 1.05 can cover above 95% pixels of local regions.

**Fig. 5:**
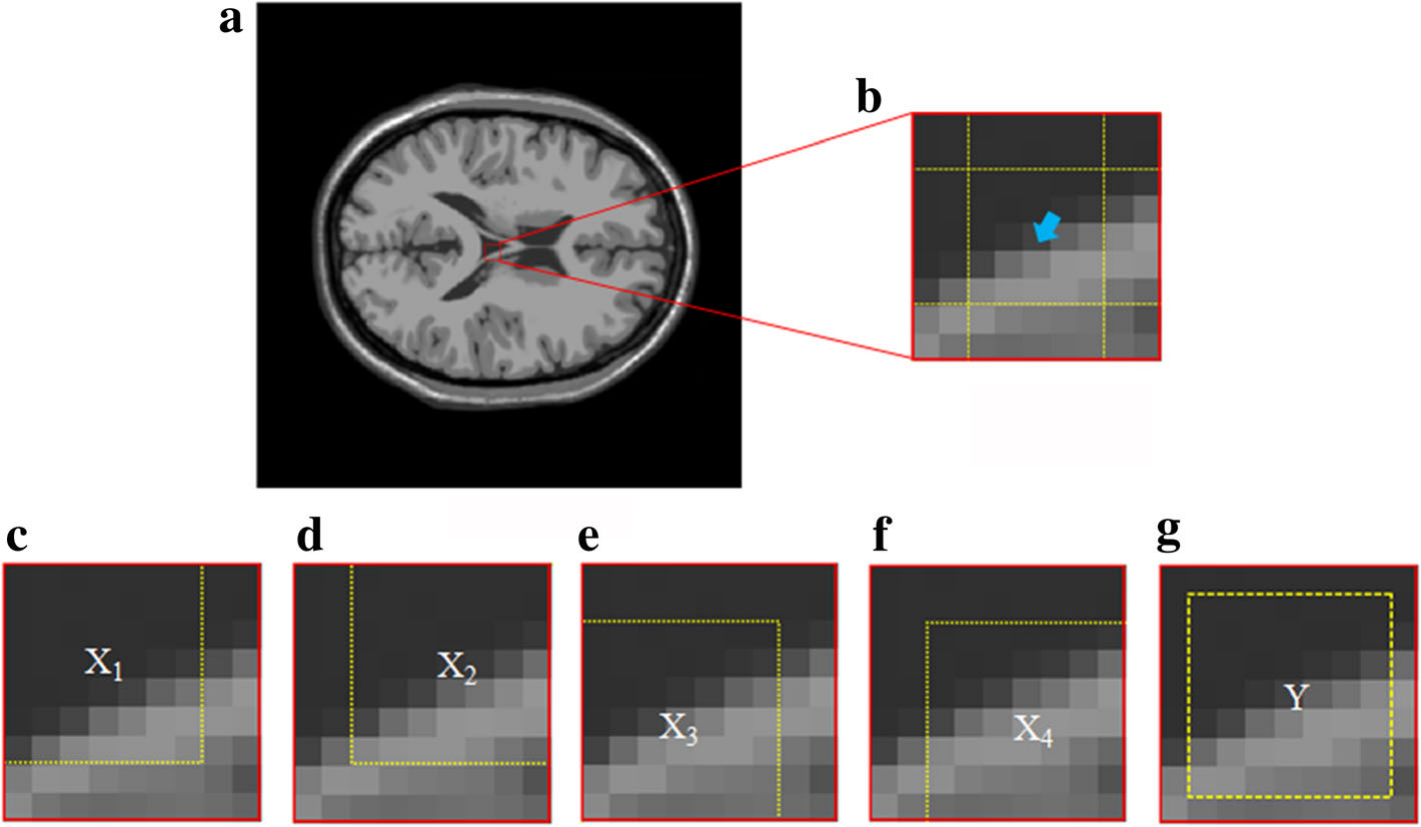
An illustration of ∑_*j* = 1_^4^ *b*_*j*_ ≈ 1. **a** A synthetic image of size 256 × 256 in which the red solid wireframe draws out a local region of size 9 × 9; **b** Repeated pixels of each **x**_*j*_ and **y** are indicated by an arrow; Collections of all of pixels from **x**_*j*_ and **y** were displayed in (**c**-**g**) respectively

An explanation on why sum of weights is nearly 1 is given. As shown in Fig. 5a, the red solid wireframe indicates the local region of size 9 × 9. Inside this region, all upper left pixels **x**_*j*_(*j* = 1) come from the pixels in the marked region X_1_ in Fig. 5c. In the same way, the upper right **x**_*j*_(*j* = 2), bottom left **x**_*j*_(*j* = 3) and bottom right **x**_*j*_(*j* = 4) will be from *X*_2_, X_3_ and X_4_, respectively. Meanwhile, the central pixels **y** are extracted from the marked region in Fig. 5g. Thus, we can see abundantly repeated pixels (suggestion of an arrow in Fig. 5b) are in these vectors. When the repeated pixels account for a big proportion in the region with a sufficiently large size, the sum of pixel value in each vector comes near to one another, implying that ∑_*i* = 1_^*n*^ *x*_*i*,1_ = ∑_*i* = 1_^*n*^ *x*_*i*,2_ = ∑_*i* = 1_^*n*^ *x*_*i*,3_ = ∑_*i* = 1_^*n*^ *x*_*i*,4_. Then, one can infer that sum of weights can be nearly 1 in Eq. (9).

#### Shared weights in multi-contrast images

In this section, the case where the weights in one image are close to those of another contrast image will be analyzed.

**Fig. 6:**
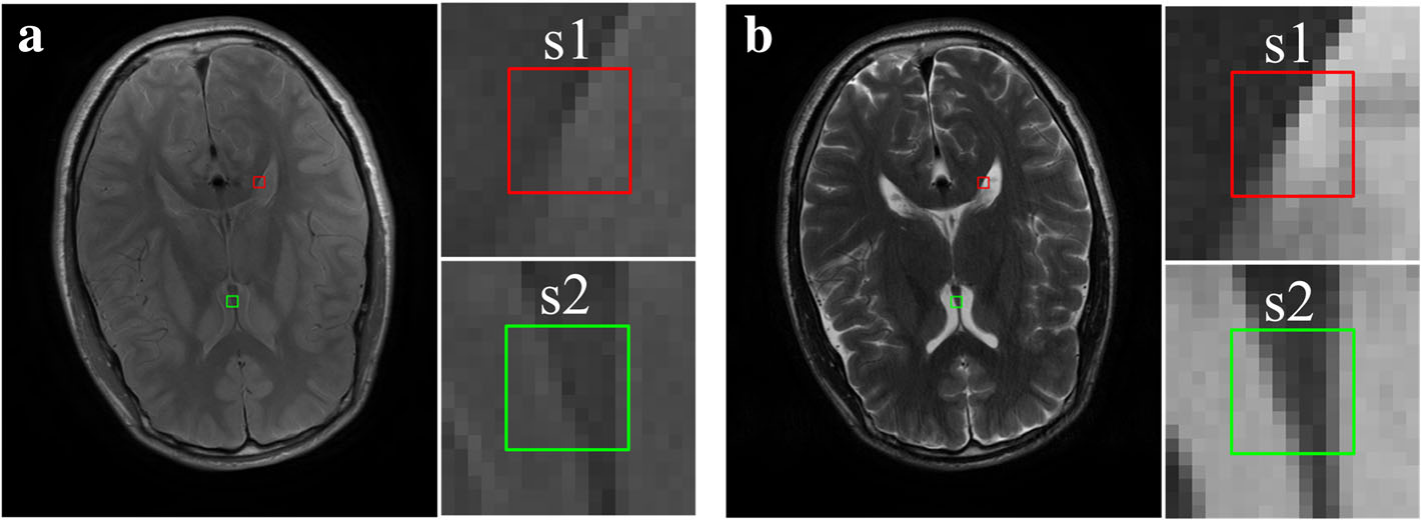
Regression weights within local regions of T1-weighted and T2-weighted MRI images. **a** is the T1-weighted image; **b** is the T2-weighted image. Two pairs of image region of size 9 × 9 (enclosed in wireframes, marked as S1 and S2) are extracted from (**a**) and (**b**). Note: The data are acquired on a 3 T SIMENS scanner

Regression weights within a small region are determined mostly by the main edge direction in it. These weights are mainly estimated from similar image patches located on edges. In the sense of least square, the influence of contrast on weights regression is very limited since multiplication of a linear system of equations by a constant factor does not change its solution. For example, in Fig. 6a and b, one can see that corresponding regions in the T1 image (Fig. 6a) and T2 images (Fig. 6b) generate similar weights (Table 4).

**Table 4:** Regression weights for T1-weighted and T2-weighted images

Source images	Regression weights **b**	
	S1	S2
T1	[−0.10; 0.56; 0.71; −0.16]	[0.76; −0.26; −0.06; 0.53]
T2	[−0.04; 0.54; 0.60; −0.07]	[0.76; −0.26; −0.19; 0.65]

The mathematical analysis on weights is simplified as listed below:

Weights error meets the following equation (see the derivation of Eq. (A.6) in the Additional file 1: Appendix for details)

10 $$ {\left\Vert \tilde{\mathbf{b}}-\mathbf{b}\right\Vert}_2\le {\left\Vert {\tilde{\mathbf{X}}}^{+}\left(\mathbf{y}-\mathbf{X}\mathbf{b}\right)\right\Vert}_2+{\left\Vert {\tilde{\mathbf{X}}}^{+}\left(\mathbf{d}-\mathbf{C}\mathbf{b}\right)\right\Vert}_2. $$

Regression weights are estimated by continually sampling 3 × 3 patches in a 9 × 9 region, and each patch is composed of one pixel *y*_*i*_ and its 4 neighbors *x*_*i*,*j*_(*j* = 1, 2, 3, 4) along diagonal directions. Consequently, the vector **y** = [*y*_1_, ⋯, *y*_*i*_, ⋯, *y*_49_]^*T*^ ∈ *ℝ* ^49^ denotes pixels in this region and the matrix **X** = [**x**_1_, ⋯, **x**_*i*_, ⋯, **x**_49_]^*T*^ ∈ *ℝ* ^49 × 4^ stands for all neighboring pixels around those pixels of **y**. Here, **X** (or $\tilde{\mathbf{X}}$) is the column-full-rank matrix and their generalized inversions are represented by **X** ^+^ and ${\tilde{\mathbf{X}}}^{+}$, respectively. In addition, there are the vector **d** = **ỹ** − **y** ∈ *ℝ* ^49^ and the matrix $\mathbf{C}=\tilde{\mathbf{X}}-\mathbf{X}\in {\mathbb{R}}^{49\times 4}$.

**Fig. 7:**
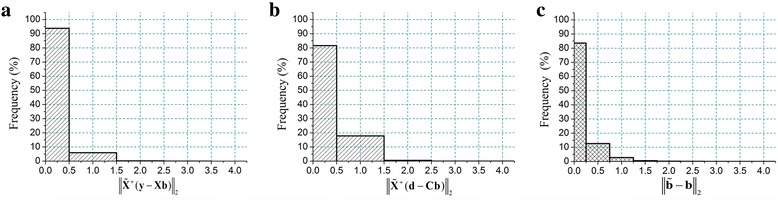
Error of regression weights on real MRI images. Weights are estimated within each pair of regions at multi-contrast images. The vertical axis represents the percentage that estimation values lies in the range of the horizontal axis. **a**-**c** list the frequency that ${\left\Vert {\tilde{\mathbf{X}}}^{+}\left(\mathbf{y}-\mathbf{X}\mathbf{b}\right)\right\Vert}_2$, ${\left\Vert {\tilde{\mathbf{X}}}^{+}\left(\mathbf{d}-\mathbf{C}\mathbf{b}\right)\right\Vert}_2$ and ${\left\Vert \tilde{\mathbf{b}}-\mathbf{b}\right\Vert}_2$ occurs in the range of the horizontal axis in (**a**-**c**), respectively

We measure the right hand of Eq. (10) on real MRI images at different regions and observations are summarized in Fig. 7. First, most of ${\left\Vert {\tilde{\mathbf{X}}}^{+}\left(\mathbf{y}-\mathbf{X}\mathbf{b}\right)\right\Vert}_2$ are very close to 0 (Fig. 7a). Besides, most of ${\left\Vert {\tilde{\mathbf{X}}}^{+}\left({\mathbf{d}}^{\mathbf{\prime}}-{\mathbf{C}}^{\mathbf{\prime}}\mathbf{b}\right)\right\Vert}_2$ is close to 0 (Fig. 7b). Therefore, the left hand of Eq. (10) approaches to 0 in most regions, implying that $\mathbf{b}\approx \tilde{\mathbf{b}}$. This conclusion is confirmed in Fig. 7c, showing that almost 84% of ${\left\Vert \tilde{\mathbf{b}}-\mathbf{b}\right\Vert}_2$ lies in small values (in the range [0, 0.25]) for the tested multi-contrast MRI images.

## Results and discussions

In experiments, we verify our approach on realistic T1-weighted and T2-weighted brain MRI images. 256 × 256 T1 and T2 HR images in Fig. 9 are from Philips Company. The T1 (TR = 170 ms, TE = 3.9 ms) and T2 (TR = 3000 ms, TE = 80 ms) datasets are acquired with Fast Field Echo (FFE) sequence (FOV = 230 × 230 mm^2^, slice thickness = 5.0 mm). The FFE sequence is a steady state gradient echo sequence acquired from Philips Company. The name of FFE is the trade name in Philips Company, and its common name is SSFP-FID. Corresponding trade name of this sequence in Siemens Company is FISP and in GE Company is GRASS. Figure 10 and Fig. 11 are acquired at a 3 T Siemens Trio Tim MRI scanner using a turbo spin echo sequence (FOV = 230 × 187 mm^2^, slice thickness = 5.0 mm) and the matrix size of T1 (TR = 2000 ms, TE = 9.7 ms) and T2 (TR = 5000 ms, TE = 97 ms) HR images is 384 × 324.

### Super-resolution experiments

**Fig. 8:**
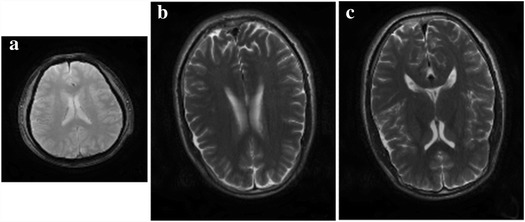
Input images. The original HR T2-weighted vision of (**a**) is acquired on a 3 T Philips scanner; Original HR T2-weighted visions of (**b**) and (**c**) are acquired on a 3 T SIMENS scanner

Before conducting the interpolation simulation, HR images are first blurred by 3 × 3 Gaussian smooth filter with standard deviation 0.5 and then down-sampled by a factor of 2 to obtain their LR versions as listed in Fig. 8. The LR image will be expanded as large as the HR reference by using the basic nearest neighbor interpolation. Then these interpolated pixels will be updated using the proposed approach.

The proposed method aims to recover edge details of LR brain image. We only borrow the weight from another HR contrast image if a pixel in the expanded LR image is located on an edge. In our work, a pixel is declared to be an edge pixel if the local variance within the nearest neighbors is above a given threshold (=0.0001, under the condition of intensities of images are all normalized between 0 and 1). We set the same value of the threshold in all experiments. Although, in some locations, it is not enough to satisfy the property of weights similarity, they only take a very small proportion of the total and are not processed specially in the proposed method.

The proposed approach is compared with the bicubic method, NEDI [18], and CGI [12]. The CGI method is used to guide the interpolation process by conducting directional filtering and achieves superior results compared to traditional interpolation techniques and other state-of-the-art edge-guided image interpolation methods. Three objective criteria, Peak Signal-to-Noise Ratio (PSNR), the Structural Similarity (SSIM) [23] and the relative *l*_2_ norm error (RLNE), are used to quantitatively measure the supper-resolution performance. The higher PSNR indicates that the reconstructed pixel value is more consistent to the original HR image and the higher SSIM implies better image structures are preserved. Also, the lower RLNE implies better consistency to the original HR image.

For the proposed method, we set the region size as 9 × 9. Within each region, 3 × 3 size patches with 1-pixel-width overlap between adjacent patches is set to maximally explore the statics in the local region. These are typical settings in the original NEDI method and works well for tested images. For CGI, default parameters are used in the shared source code.

**Fig. 9:**
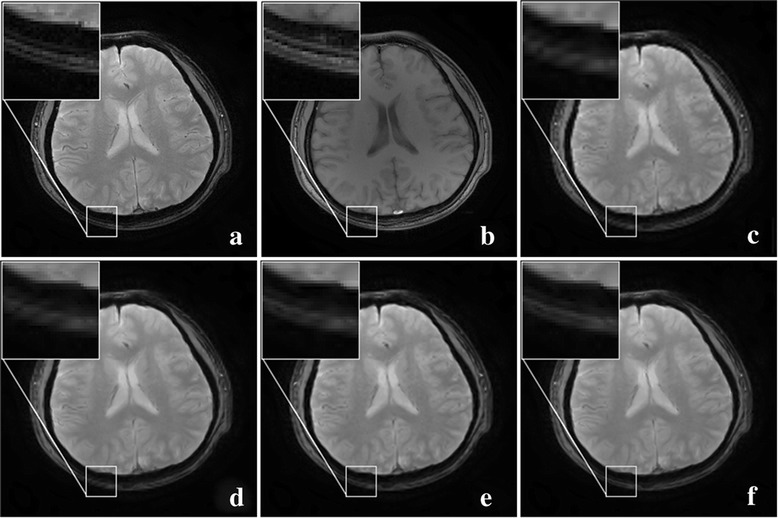
One pair of T1 and T2 MRI images acquired on 3 T Philips scanner. **a** HR of T2; **b** HR of T1; **c** the bicubic; **d** NEDI; **e** CGI; **f** the proposed method

First pair of images in Fig. 9 clearly show the advantage of employing the statistical information from a HR image in another contrast. Blocky artifacts in Fig. 9c are obviously generated using the classic bicubic method. The NEDI method outperforms the bicubic method since sharper edges are observed in Fig. 9d. The CGI method recovers brain boundaries in Fig. 9e much better than NEDI. Most promising edges (Fig. 9f) are produced by the proposed approach.

**Fig. 10:**
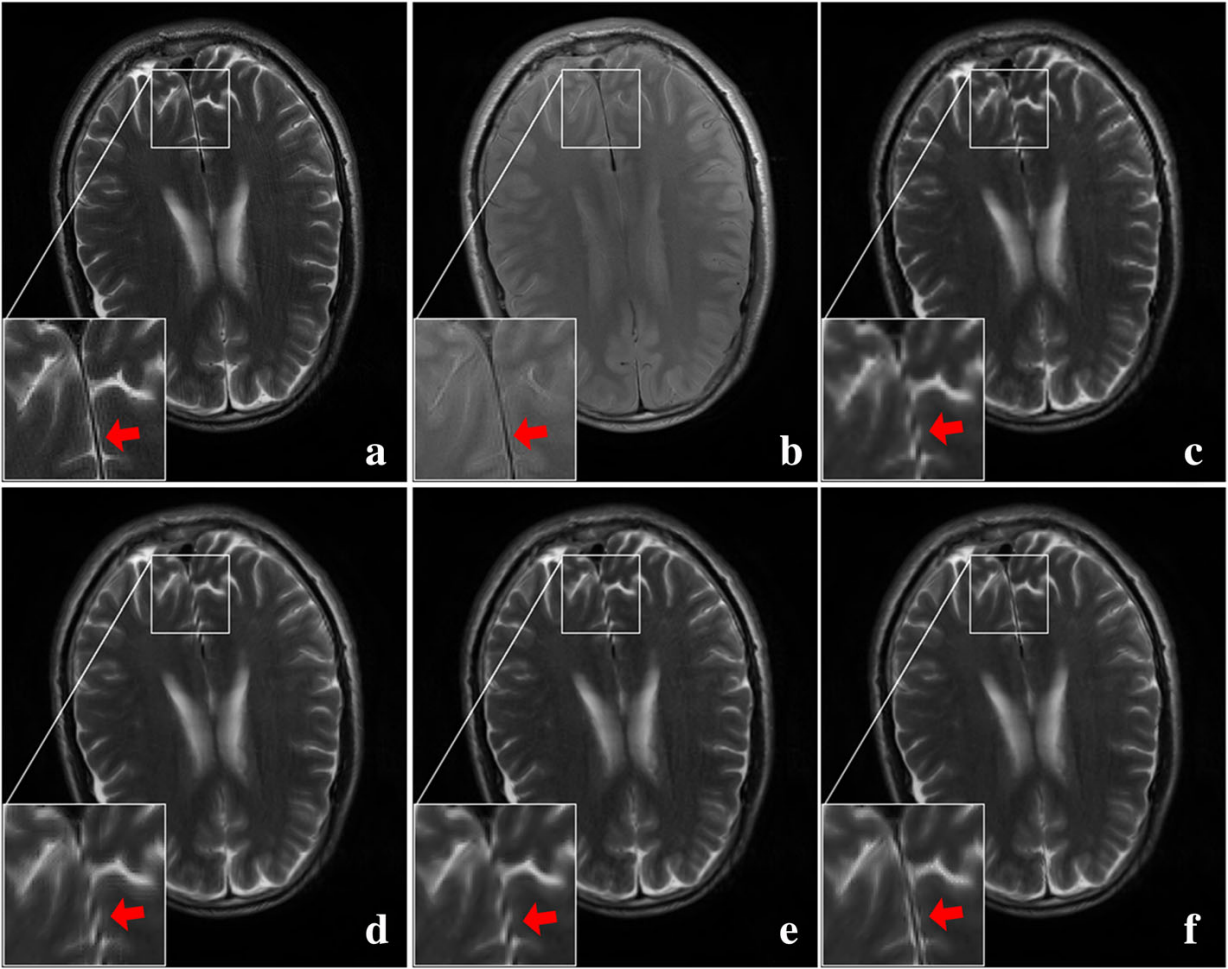
One pair of T1 and T2 MRI images acquired on 3 T Siemens scanner. **a** HR of T2 image; **b** HR of T1 image; **c**-**f** are super-resolved images using the bicubic, NEDI, CGI, and the proposed method, respectively

**Fig. 11:**
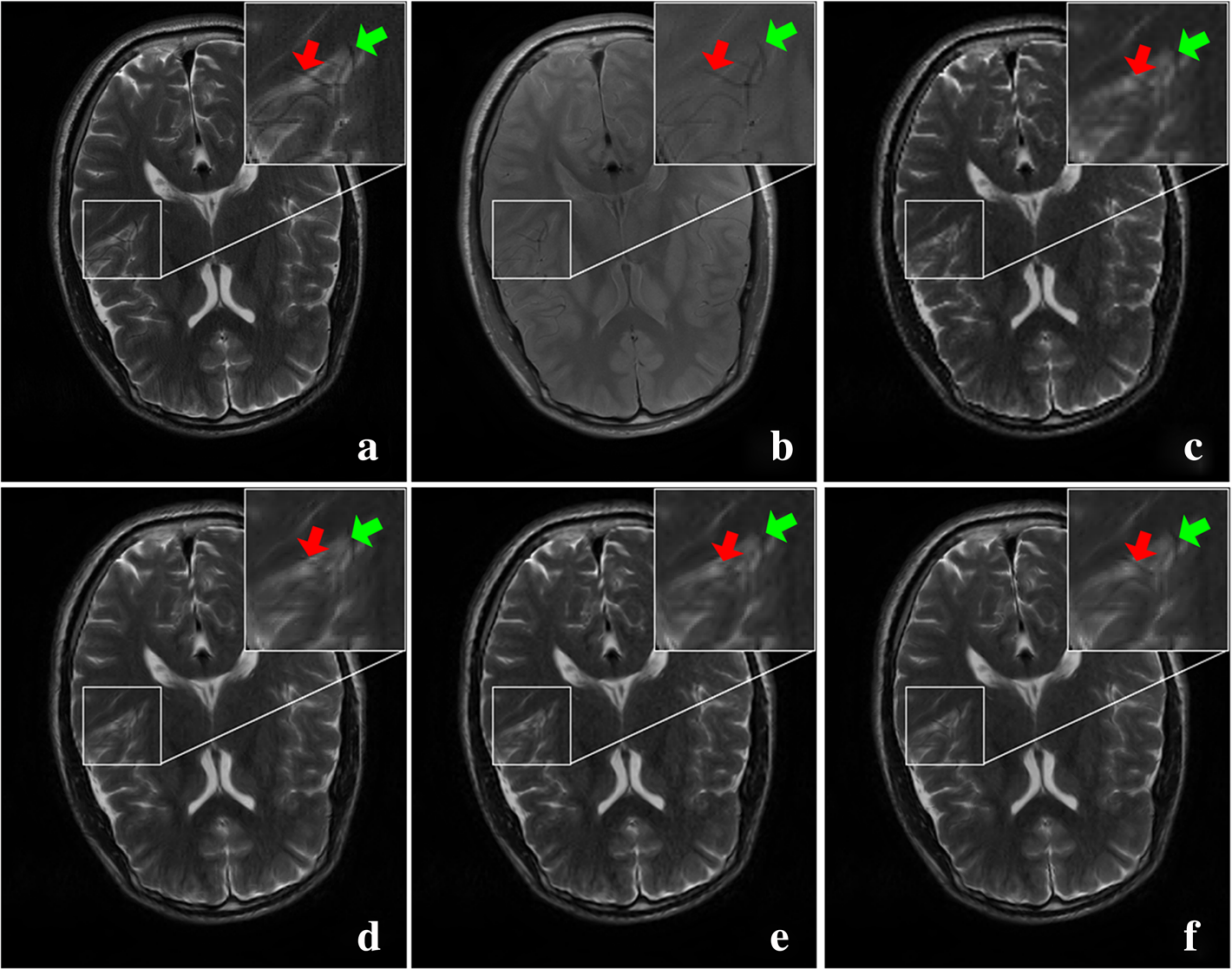
Another pair of T1 and T2 MRI images acquired on 3 T Siemens scanner. **a** HR of T2 image; **b** HR of T1 image; **c-f** are super-resolved images using the bicubic, NEDI, CGI, and the proposed method, respectively

For another two pairs of images acquired on a 3 T MRI scanner in Figs. 10 and 11, it can also be observed that there are many artifacts around some edges (seeing arrows) by the bicubic method. Such artifacts can be reduced by interpolation of using NEDI and CGI, and the proposed method still produces most faithful edges.

The CGI obtains higher PSNR and SSIM and lower RLNE than both NEDI and the classic bicubic. The best objective criteria are achieved by the proposed approach as listed in Table 5. These criteria are consistent to the image quality analyzed above.

**Table 5:** PSNR/SSIM/RLNE evaluation for different methods

Images	The bicubic	NEDI	CGI	The proposed
Fig. 9	28.55/0.8738/0.1159	31.55/0.9117/0.0820	31.79/0.9168/0.0798	**31.90/0.9190/0.0788**
Fig. 10	30.67/0.9121/0.1532	33.12/0.9347/0.1155	33.73/0.9396/0.1077	**33.89/0.9400/0.1057**
Fig. 11	29.39/0.8986/0.1767	32.60/0.9282/0.1221	33.09/0.9341/0.1155	**33.15/0.9345/0.1146**
Fig. A1	29.38/0.9067/0.1800	31.26/0.9389/0.1451	31.81/0.9446/0.1362	**32.17/0.9466/0.1306**
Fig. A2	28.50/0.8849/0.1819	30.74/0.9196/0.1405	31.21/0.9260/0.1331	**31.32/0.9262/0.1314**

### Sensitivity to the Misregistration

To evaluate how the misalignment affects the accuracy of the reconstruction result, we shift reference images along different directions (e.g., slant, anti-slant, vertical and horizontal) by a certain amount of pixels [5]. First, we compute the evaluation criteria of CGI and the proposed method using the ground truth HR image and the interpolated HR images; Second, each number in Table 6 is obtained by subtracting the evaluation criteria of the CGI from of the proposed method and is referred as “the improvement of the PSNR or SSIM or RLNE”. The positive number means that the proposed method outperforms CGI method, implying better tolerance of image misregistration. From Table 6, one can see that, under 1 to 2-pixel-shift, the proposed method holds advantage over CGI.

**Table 6:** Improvements of PSNR/SSIM/RLNE compared with CGI method showed in Fig. 11

Pixels to move	Directions of move			
	Slant	Anti-slant	Vertical	Horizontal
0	+0.06/+0.0004/+0.0009			
1	+0.10/+0.0004/+0.0014	**+0.05/+0.0003/+0.0007**	**+0.04/+0.0002/+0.0006**	+0.07/+0.0006/+0.0010
2	**+0.08/+0.0001/+0.0011**	−0.05/−0.0007/−0.0006	−0.0003/−0.0001/0	**+0.03/0/+0.0004**
3	+0.06/−0.0006/+0.0009	−0.39/−0.0033/−0.0053	−0.05/−0.0005/−0.0005	−0.25/−0.0022/−0.0034
4	−1.04/−0.0081/−0.0145	−1.57/−0.0111/−0.0229	−0.51/–0.0039/−0.0069	−1.29/−0.0092/−0.0184

One slice of the brain image in Fig. 11 is used in simulation.

### Structural distinction in T1 and T2

In MRI, T1 and T2 images have some distinct signal intensity that may cause structural distinctions appeared. For example, a structure can be visible clearly in the T2 image and is embodied too little in the T1 image (Fig. 10a and b, arrow B), or, in turn, a structure can be visible in T1 image and is embodied too little in the T2 image (Fig. 10a and b, arrow A). These distinct structures may be lesions or normal organisms but are not ghosts. This is normal phenomenon in MRI.

**Fig. 12:**
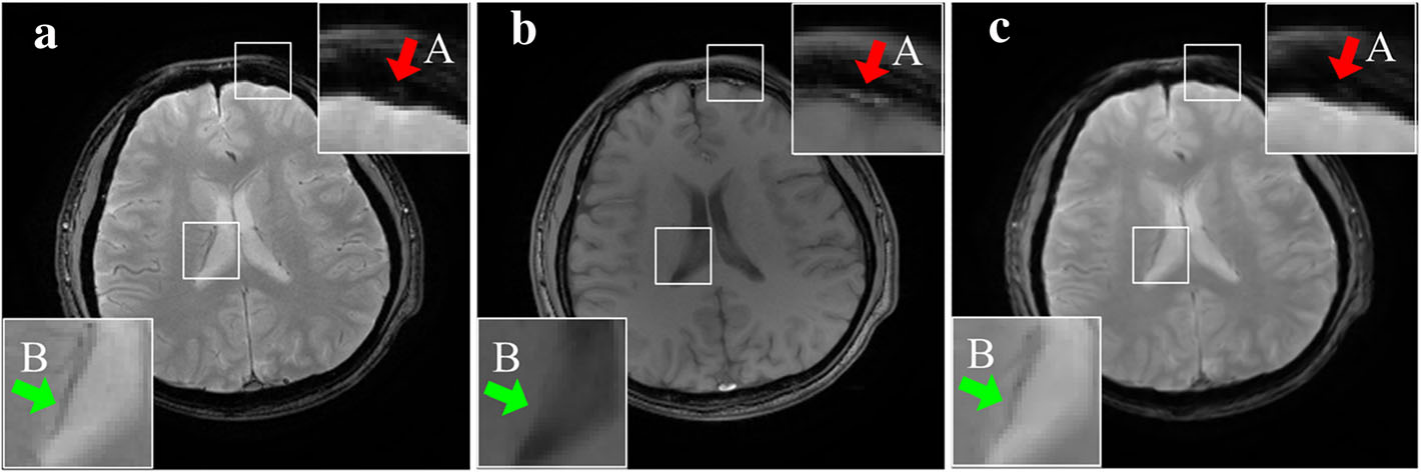
Super-resolution of structural distinctions. **a** The HR of T2 image (the ground-truth); **b** The HR T1 image (the reference); **c** The proposed

As discussed in Super-resolution experiments, we know estimated weights are nearly invariant to image contrasts. Therefore, the super-resolution still can work decently. Fig. 12 demonstrates the proposed method produces structures consistent with the ground-truth. For example, if a structure is observed on the reference but not on the ground-truth HR image, the proposed approach will not introduce the structure into the reconstruction (Fig. 12, arrow A). Other structures, which are found on the ground-truth image but not on the reference, can be recovered faithfully (Fig. 12, arrow B). These recovered structures are not reproduced correctly as well as in the ground-truth image, and appear blurrier than its vision in the ground-truth image.

### Image denoising

We agree that the noise is not obviously presented in the tested brain imaging datasets. But the proposed method has the ability to suppress noise since regression weights are estimated according to the least square rule, which intrinsically has the ability to suppress noise.

**Fig. 13:**
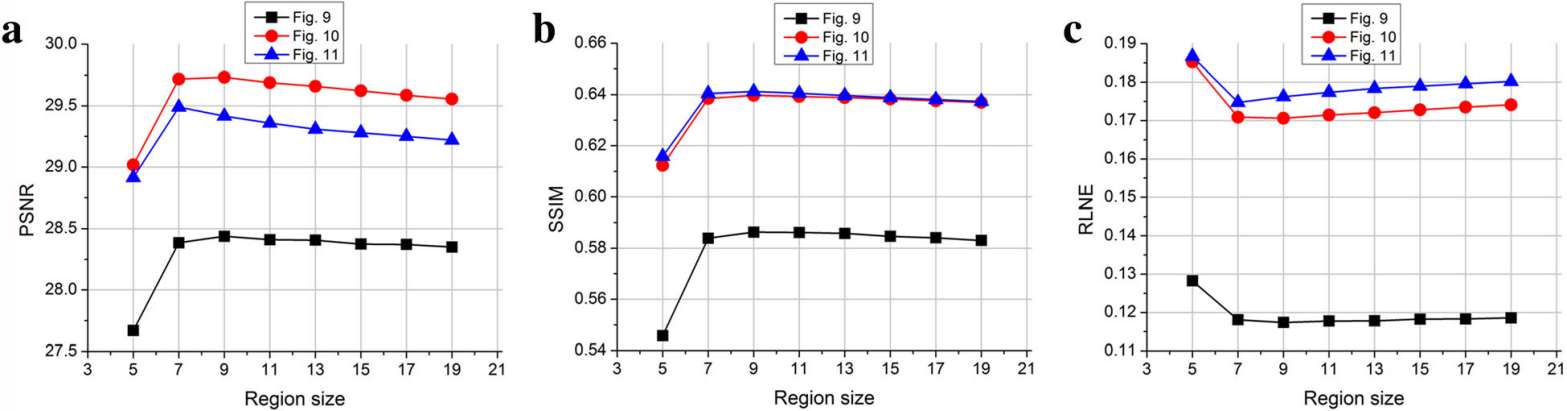
Effects of noise with various region sizes. Note: To simulate the 3% Rician noise, the zero mean Gaussian noise are added to real and imaginary parts of T2-weighted images, respectively. (**a**-**c**) are PSNR, SSIM and RLNE, respectively

To further elaborate the noise removal, the noise at common levels (1, 3 and 5% of the maximum intensity) [24, 25] is added into the ground-truth image. Results of 3% noise in Fig. 13 imply that reducing the region size to 5 × 5 or increase to be larger than 9 × 9 will reduce the PSNR, SSIM and increase the reconstruction error, RLNE. Therefore, a region size of 7 × 7 or 9 × 9 is suggested to optimally suppress the noise. For other noise levels, trend curves of objective criteria are similar with Fig. 13 and come to the same conclusion.

We also comment that if the serious noise that may injury the interpolation result, noise removal before the interpolation should be accomplished. This is beyond the scope of this work and we leave this as the future work.

### Computation time

Our method is implemented with MATLAB on a personal computer with Dual-Core CPU 3.00GHz and 2GB memory. The computation time of the proposed method is very close to NEDI, and costs around 10 s.

## Conclusions

An MRI image super-resolution approach is proposed to employ the statistical information retrieved from another contrast MRI image that shares similar anatomical structures. It is found that local regression weights are very similar among multi-contrast MRI images. This property is analyzed with comprehensive mathematics and experimental evidence. Experiment results demonstrate that the image quality of the low-resolution image can be truly improved if the contrast-invariant weight is borrowed from the high resolution image of another contrast. In the future, we plan to further improve the sharpness of edges and textures by utilizing sparse representation [26–29] and local geometric directions [30–32]. The code of this work is available at http://www.quxiaobo.org/project/MultiContrastMRI/Toolbox_MultiContrastMRI_Superresolution.zip.

### Highlights

- Multi-contrast MR images share similar anatomical structures, e.g., the T1-weighted and the T2-weighted images.
- Regression weights are found to be similar among multi-contrast images.
- Comprehensive mathematics and numerical experiments are presented trying to analyze the weights-similarity property.
- Regression weights are learnt from another contrast high-resolution MRI image.
- An MRI image super-resolution approach using local regression weights is proposed.
- Compared with classic state-of-the-art interpolation techniques, the performance of the proposed method is remarkably improved.

## Additional file

Additional file 1: Detailed formula derivations, analysis of regression weight in completely opposite contrast images, and more super-resolved MRI images. (DOCX 1215 kb)

## Abbreviations

CGI: Contrast-guided interpolation
HR: High-resolution
LR: Low-resolution
MRI: Magnetic resonance imaging
NEDI: New edge-directed interpolation
POCS: Projection onto convex sets
PSNR: Peak signal-to-noise ratio
RLNE: Relative L2 norm error
SSIM: Structural similarity

## References

[1] Peled S, Yeshurun Y (2001). Superresolution in MRI: application to human white matter fiber tract visualization by diffusion tensor imaging. Magn Reson Med.

[2] Scherrer B, Gholipour A, Warfield SK (2012). Super-resolution reconstruction to increase the spatial resolution of diffusion weighted images from orthogonal anisotropic acquisitions. Med Image Anal.

[3] Poot DHJ, Jeurissen B, Bastiaensen Y, Veraart J, Van Hecke W, Parizel PM, Sijbers J (2013). Super-resolution for multislice diffusion tensor imaging. Magn Reson Med.

[4] Kornprobst P, Peeters R, Nikolova M, Deriche R, Ng M, Van Hecke P (2003). A superresolution framework for fMRI sequences and its impact on resulting activation maps. Med Image Comput Computering-Assisted Intervention (MICCAI’03) (Montreal, Canada).

[5] Manjón JV, Coupé P, Buades A, Collins DL, Robles M (2010). MRI Superresolution using self-similarity and image priors. Int J Biomed Imaging.

[6] Yang B, Yuan M, Ma Y, Zhang J, Zhan K (2015). Local sparsity enhanced compressed sensing magnetic resonance imaging in uniform discrete curvelet domain. BMC Med Imaging.

[7] Qu X, Hou Y, Lam F, Guo D, Zhong J, Chen Z (2014). Magnetic resonance image reconstruction from undersampled measurements using a patch-based nonlocal operator. Med Image Anal.

[8] Wong A, Liu C, Wang X, Fieguth P, Bie H (2015). Homotopic non-local regularized reconstruction from sparse positron emission tomography measurements. BMC Med Imaging.

[9] Wang TT, Cao L, Yang W, Feng QJ, Chen WF, Zhang Y (2015). Adaptive patch-based POCS approach for super resolution reconstruction of 4D-CT lung data. Phys Med Biol.

[10] Ding HJ, Gao H, Zhao B, Cho HM, Molloi S (2014). A high-resolution photon-counting breast CT system with tensor-framelet based iterative image reconstruction for radiation dose reduction. Phys Med Biol.

[11] Huang JH, Guo L, Feng QJ, Chen WF, Feng YQ (2015). Sparsity-promoting orthogonal dictionary updating for image reconstruction from highly undersampled magnetic resonance data. Phys Med Biol.

[12] Wei Z, Ma KK (2013). Contrast-guided image interpolation. IEEE Trans Image Process.

[13] Greenspan H (2009). Super-resolution in medical imaging. Comput J.

[14] Mark AB, Richard CS. MRI Basic Principles and Applications. Wiley-Liss 2003.

[15] Rousseau F. Brain hallucination. In Prceedings of the European Conference on Computer Vision (ECCV'08) (New York, USA). 2008; Part 1. p. 497–508.

[16] Rousseau F (2010). A non-local approach for image super-resolution using intermodality priors. Med Image Anal.

[17] Jafari-Khouzani K (2014). MRI upsampling using feature-based nonlocal means approach. IEEE Trans Med Imag.

[18] Li X, Orchard MT (2001). New edge-directed interpolation. IEEE Trans Image Process.

[19] Timofte R, De Smet V, Van Gool L. Anchored neighborhood regression for fast example-based super-resolution. IEEE Int Conf Comput Vis (ICCV’13) (Sydney, Australia). 2013:1920–7.

[20] Yang CY, Yang MH. Fast direct super-resolution by simple functions. IEEE Int Conf Comput Vis (ICCV’13) (Sydney, Australia). 2013:561–8.

[21] Dai D, Timofte R, Van Gool L (2015). Jointly optimized regressors for image super-resolution. Comput Graph Forum.

[22] Cocosco CA, Kollokian V, Kwan RKS, Evans AC (1997). BrainWeb: online interface to a 3D MRI simulated brain database. Neuroimage.

[23] Wang Z, Bovik AC, Sheikh HR, Simoncelli EP (2004). Image quality assessment: from error visibility to structural similarity. IEEE Trans Image Process.

[24] Manjo’n JV, Caballero JC, Lull JJ, Martı’ GG, Bonmatı’ LM, Robles M (2008). MRI denoising using Non-local means. Med Image Anal.

[25] Gudbjartsson H, Patz S (1995). The Rician distribution of noisy MRI data. Magn Reson Med.

[26] Ravishankar S, Bresler Y (2011). MR image reconstruction from highly undersampled k-space data by dictionary learning. IEEE Trans Med Imaging.

[27] Ravishankar S, Bresler Y (2015). Efficient blind compressed sensing using sparsifying transforms with convergence guarantees and application to magnetic resonance imaging. SIAM J Imaging Sci.

[28] Liu Y, Zhan Z, Cai JF, Guo D, Chen Z, Qu X (2016). Projected iterative soft-thresholding algorithm for tight frames in compressed sensing magnetic resonance imaging. IEEE Trans Med Imaging.

[29] Zhan Z, Cai JF, Guo D, Liu Y, Chen Z, Qu X (2016). Fast multiclass dictionaries learning with geometrical directions in MRI reconstruction. IEEE Trans Biomed Eng.

[30] Qu X, Guo D, Ning B, Hou Y, Lin Y, Cai S, Chen Z (2012). Undersampled MRI reconstruction with patch-based directional wavelets. Magn Reson Imaging.

[31] Ning B, Qu X, Guo D, Hu C, Chen Z (2013). Magnetic resonance image reconstruction using trained geometric directions in 2D redundant wavelets domain and non-convex optimization. Magn Reson Imaging.

[32] Lai Z, Qu X, Liu Y, Guo D, Ye J, Zhan Z, Chen Z (2016). Image reconstruction of compressed sensing MRI using graph-based redundant wavelet transform. Med Image Anal.

