# Supplementary material for: Multi-contrast brain magnetic resonance image super-resolution using the local weight similarity
Source: BMC Med Imaging. 2017 Jan 17;17:6. doi: 10.1186/s12880-016-0176-2 (PMC5240324; doi:10.1186/s12880-016-0176-2)
Supplement: Additional file 1: — Detailed formula derivations, analysis of regression weight in completely opposite contrast images, and more super-resolved MRI images. (DOCX 1215 kb) [file 12880_2016_176_MOESM1_ESM.docx]

**Appendix A (Fig. A1, Fig. A2, Fig. A3 and Fig. A4)**

Multi-Contrast Brain Magnetic Resonance Image Super-Resolution Using the Local Weight Similarity

Hong Zheng^1, 2^, Xiaobo Qu^1*^, Zhengjian Bai^3^, Yunsong Liu^1^, Di Guo^4^, Jiyang Dong^1^, Xi Peng^5^, Zhong Chen^1*^

**Author details**

^1^Department of Electronic Science, Xiamen University, Xiamen 361005, China;

^2^School of Computer Science and Engineering, Key Laboratory of Intelligent Processing of Image and Graphics, Guilin University of Electronic Technology, Guilin 541004, China;

^3^School of Mathematical Sciences, Xiamen University, Xiamen 361005, China

^4^School of Computer and Information Engineering, Fujian Provincial University Key Laboratory of Internet of Things Application Technology, Xiamen University of Technology, Xiamen 361024, China

^5^Paul C. Lauterbur Research Centre for Biomedical Imaging, Shenzhen Institutes of Advanced Technology, Shenzhen, Guangdong 518055, China.

Hong Zheng, Email: 458436419@qq.com;

Xiaobo Qu, Email: [quxiaobo@xmu.edu.cn](mailto:quxiaobo@xmu.edu.cn); (Corresponding author)

Zhengjian Bai, Email: zjbai@xmu.edu.cn;

Yunsong Liu, Email: yunsongliu@qq.com;

Di Guo, Email: guodi@xmut.edu.cn;

Jiyang Dong, Email: jydong@xmu.edu.cn;

Xi Peng, Email: xi.peng@siat.ac.cn;

Zhong Chen, Email: [chenz@xmu.edu.cn](mailto:chenz@xmu.edu.cn). (Corresponding author)

**Regression Weights Errors Using Multi-contrast Images**

We conduct the following mathematical derivation for shared weights in multi-contrast images. Let $\mathbf{b}$ and $\tilde{\mathbf{b}}$ be optimal weight vectors for two contrast images in the sense of least squares. Then

$\mathbf{b}=\mathbf{X}^{+}\mathbf{y} , \tilde{\mathbf{b}}=\tilde{\mathbf{X}}^{+}\tilde{\mathbf{y}} .$ (A.1)

where $\mathbf{y}$ (or $\tilde{\mathbf{y}}$) is a vector which contain pixels in the local region, and $\mathbf{X}$ (or $\tilde{\mathbf{X}}$) is the column-full-rank matrix, in which each row is composed of four nearest neighbors along the two diagonal directions around the pixel of the corresponding row of $\mathbf{y}$ (or $\tilde{\mathbf{y}}$). Here, $\mathbf{X}^{+}$ and $\tilde{\mathbf{X}}^{+}$ represent the generalized inversion of $\mathbf{X}$ and $\tilde{\mathbf{X}}$, respectively.

In addition, we use $\mathbf{h}$ to represent the error of regression weights according to

$\mathbf{h}=\tilde{\mathbf{b}}-\mathbf{b}.$ (A.2)

Let us suppose that and , we get

$\tilde{\mathbf{X}}=\mathbf{X}+\mathbf{C}, \tilde{\mathbf{y}}=\mathbf{y}+\mathbf{d}.$ (A.3)

Then substituting Eq. (A.1) and Eq. (A.3) into Eq. (A.2), one obtains

$\mathbf{h}=\tilde{\mathbf{X}}^{+}(\mathbf{y}+\mathbf{d})-\mathbf{X}^{+}\mathbf{y}.$ (A.4)

By simple mathematical calculation on Eq. (A.4), we get

$\mathbf{h}=\tilde{\mathbf{X}}^{+}(\mathbf{y}-\mathbf{Xb})+\tilde{\mathbf{X}}^{+}(\mathbf{d}-\mathbf{Cb}).$ (A.5)

Therefore, the norm of regression weight errors between two contrast images is bounded by

$\left\|\mathbf{h}\right\|_{2}\leq\left\|\tilde{\mathbf{X}}^{+}(\mathbf{y}-\mathbf{Xb})\right\|_{2}+\left\|\tilde{\mathbf{X}}^{+}(\mathbf{d}-\mathbf{Cb})\right\|_{2}.$ (A.6)

**Completely Opposite Contrast Images**

Here, we will analyse a simple and excessive case. Suppose two multi-contrast images have completely opposite contrasts (Figs. A1a and A1b), the vector $\mathbf{b}$ can be found by

(A.7)

and the vector $\tilde{\mathbf{b}}$ in the opposite contrast image can be found by

$\tilde{\mathbf{b}}=\arg\min_{\tilde{b}_{j}} \{\sum_{i=1}^{49} ((1-y_{i})-\sum_{j=1}^{4} (1-x_{i,j})\tilde{b}_{j})^{2}\}.$ (A.8)

Since most of $\sum_{j=1}^{4} \tilde{b}_{j}$ are close to 1, it follows that $((1-y_{i})-\sum_{j=1}^{4} (1-x_{i,j})\tilde{b}_{j})^{2}$ is close to $(y_{i}-\sum_{j=1}^{4} x_{i,j}\tilde{b}_{j})^{2}$. Then Eq. (A.8) is approximately equal to

$\tilde{\mathbf{b}}=\arg\min_{\tilde{b}_{j}} \{\sum_{i=1}^{49} (y_{i}-\sum_{j=1}^{4} x_{i,j}\tilde{b}_{j})^{2}\}.$ (A.9)

By comparing Eq. (A.9) with Eq. (A.7), their forms are same. Therefore we can say that $\mathbf{b}$ is near to $\tilde{\mathbf{b}}$, meaning that corresponding weights in multi-contrast images are similar.

For example, in Figs. A1a and A1b, one can see that corresponding regions in completely opposite contrast images generate similar weights (Table A1).


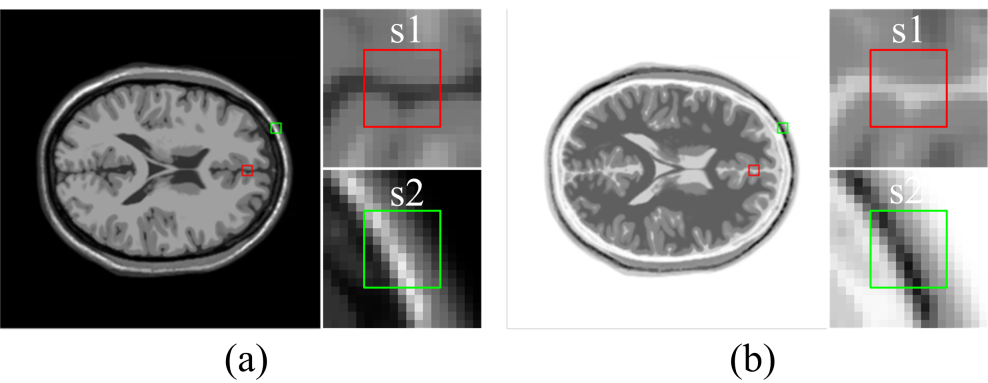


Fig. A1. Regression weights within local regions of completely opposite contrast images. (a) is an image; (b) is the opposite contrast to (a). Two pairs of image region of size 9×9 (enclosed in wireframes, marked as S1 and S2) are extracted from (a) and (b). Note: The data are acquired from the BrainWeb.

**Table A1** Regression weights within local regions of completely opposite contrast images

| Source images | Regression weights $\mathbf{b}$ | |
| --- | --- | --- |
|  | S1 S2 | |
| (a) | [0.48; 0.05; 0.06; 0.42] | [0.53; -0.11; -0.04; 0.61] |
| (b) | [0.47; 0.06; 0.06; 0.42] | [0.53; -0.11; -0.03; 0.62] |

The mathematical analysis on weights is simplified as listed below:

Considering all the regions in Figs. A1a and A1b, we draw the weights error distribution (Fig. A2) which meets Eq. (10). First, most of $\left\|\tilde{\mathbf{X}}^{+}(\mathbf{y}-\mathbf{Xb})\right\|_{2}$ are very close to 0 (Fig. A2a). Besides, most of $\left\|\tilde{\mathbf{X}}^{+}(\mathbf{d}'-\mathbf{C}'\mathbf{b})\right\|_{2}$ is close to 0 (Fig. A2b). Therefore, $\left\|\tilde{\mathbf{b}}-\mathbf{b}\right\|_{2}$ approaches to 0 in most regions, implying that $\mathbf{b}\approx\tilde{\mathbf{b}}$. This conclusion is confirmed in Fig. A2c, showing that almost 98% of $\left\|\tilde{\mathbf{b}}-\mathbf{b}\right\|_{2}$ lies in small values (in the range $[0,0.25]$) for the tested completely opposite contrast images.


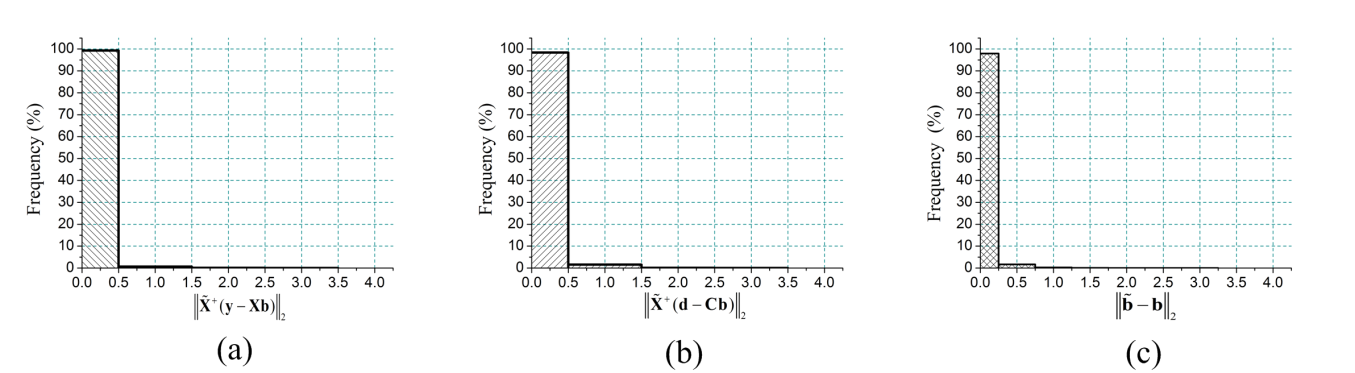


Fig. A2. Error of regression weights on completely opposite contrast images. Weights are estimated within each pair of regions at completely opposite contrast images. The vertical axis represents the percentage that estimation values lies in the range of the horizontal axis. (a)-(c) list the frequency that , $\left\|\tilde{\mathbf{X}}^{+}(\mathbf{d}-\mathbf{Cb})\right\|_{2}$ and $\left\|\tilde{\mathbf{b}}-\mathbf{b}\right\|_{2}$ occurs in the range of the horizontal axis in (a-c), respectively.

**Complements for Main Results**

Figs. A3 and A4 are taken from a 3T Siemens Trio Tim MRI scanner. The size of T1 (TR=1370ms, TE=2.29ms) and T2 (TR=3200ms, TE=411ms) HR images is 256×200 (FOV=220×220 mm^2^, slice thickness=1.0mm), and they are acquired with MPRAGE and SPACE sequence respectively.


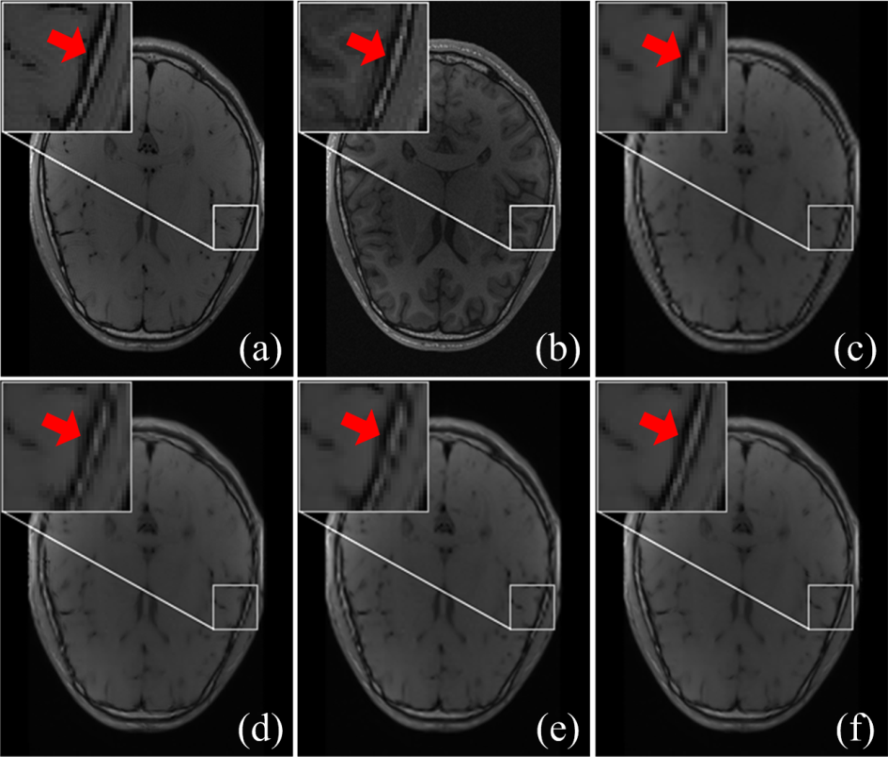


Fig. A3. One pair of T1 and T2 MRI images acquired on 3T Siemens scanner. (a) HR of T2 image; (b) HR of T1 image; (c)-(f) are super-resolved images using the bicubic, NEDI, CGI, and the proposed method, respectively.


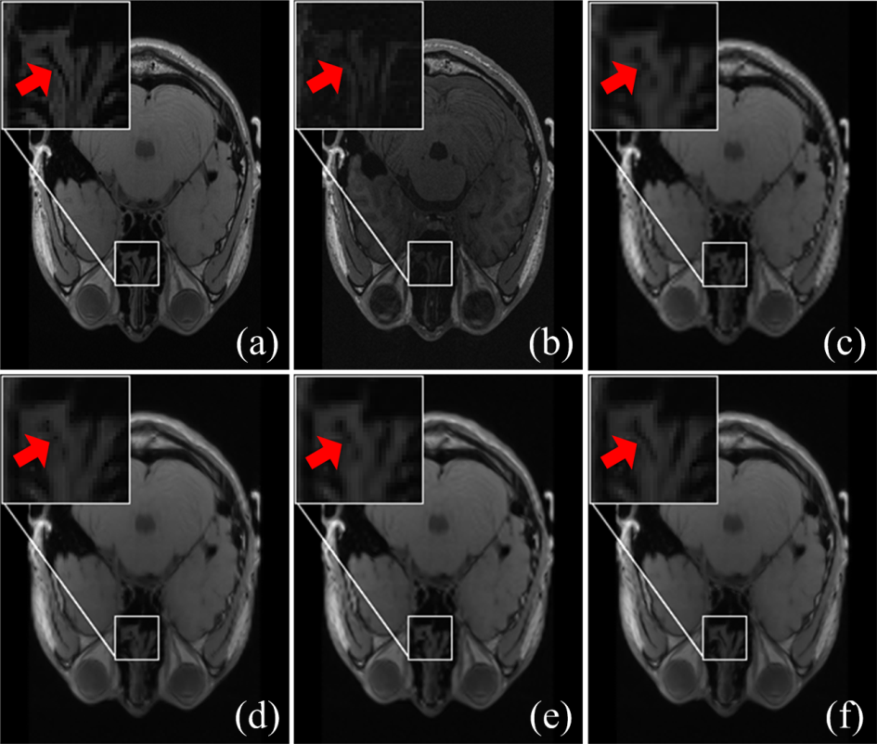


Fig. A4. Another pair of T1 and T2 MRI images acquired on 3T Siemens scanner. (a) HR of T2 image; (b) HR of T1 image; (c)-(f) are super-resolved images using the bicubic, NEDI, CGI, and the proposed method, respectively.
